# An account of American Academy of Microbiology reforms and pandemic operations

**DOI:** 10.1128/mbio.02334-23

**Published:** 2023-10-26

**Authors:** Arturo Casadevall, Peggy A. Cotter, Lynn Enquist, Timothy J. Donohue, Stefano Bertuzzi, Nguyen K. Nguyen

**Affiliations:** 1 Past Chair of the Committee of Governors of AAM, Department of Molecular Microbiology and Immunology, Johns Hopkins School of Public Health, Baltimore, Maryland, USA; 2 Past President of ASM, Department of Microbiology and Immunology, University of North Carolina at Chapel Hill, Chapel Hill, North Carolina, USA; 3 Past President of ASM and Current Governor of AAM, Department of Molecular Biology, Princeton University, Princeton, New Jersey, USA; 4 Past President of ASM, Department of Bacteriology, University of Wisconsin, Madison, Wisconsin, USA; 5 Chief Executive Officer, American Society for Microbiology, Washington, DC, USA; 6 Director of AAM, American Society for Microbiology, Washington, DC, USA

## Abstract

Change is an inevitable part of any organization if it wants to adapt and strive in a changing environment. That was what the American Academy of Microbiology (Academy) did from 2019-2023 when it transformed itself into a scientific think tank at ASM while maintaining the high standard of an honorific community of scholars. Here, we report on the recent history of the Academy and the changes that have taken place during this period. With the contribution of many thougtful leaders, the Academy refreshed its commitment to promote excellence and uphold its high values.

## EDITORIAL

Over the past 4 years, the American Academy of Microbiology undertook a program of reform to update its protocols and procedures and define a new mission. Here, we detail the reforms made and provide an account of pandemic operations with the goal of providing a record of these events.

The American Academy of Microbiology (Academy, also known as AAM) is the honorific leadership group within the American Society for Microbiology (ASM), which is one of the largest life sciences societies in the world. The mission of the ASM is to promote and advance microbial sciences. The mission of the Academy is to recognize and promote excellence in microbial sciences, provide scientific expertise, and convene scientific power in the service of science and the public. The Academy fulfills its mission by electing new fellows into the Academy, directing the ASM’s Awards Program, and convening colloquia and scientific programs. Beginning in 2019, the Academy undertook an ambitious program of reform aimed at renewing its vision, standardizing its operating procedures, increasing transparency, and better serving the ASM and the microbiology community.

### The Academy prior to 2019

The Academy was incorporated in 1955 as an independent organization responsible for accrediting microbiology training, until it merged with ASM in 1969. For several decades afterward, the Academy operated as a quasi-independent unit within ASM with its own set of bylaws focused on recognizing outstanding scientists and microbiologists by election as fellows, managing the ASM award portfolio, and holding colloquia on specialized topics that produced learned reports. However, there were problems in its operations. The Academy was governed by a Board of Governors (BoG) following bylaws and procedures that were sometimes not consistent with those of the parent organization, ASM. The fellowship election system relied on a two-tier evaluation of nominees carried out by a Committee on Elections (COE) followed by the BoG, but these two bodies sometimes disagreed on the qualification of candidates. For example, it was not uncommon for the COE to recommend a candidate that was then rejected by the Governors or for the Governors to elevate a candidate that had not been recommended by the COE. The result created a sense that some election decisions were arbitrary, fostering tension between the BoG and COE even though both groups were composed of Academy fellows of comparable stature and expertise. Adding to the governance and operational difficulties was the fact that the BoG met only once per year, resulting in little or no communication among the Governors for most of the year. By the late 2010s, it was evident that the Academy needed major reforms.

### The process of reform

Beginning in early 2019, the Academy began a review of all its protocols, procedures, and rules. The approach was to constitute small working groups to determine whether changes were needed and what those changes should be. This resulting dialogue culminated in the voting of changes, which in some cases required approval by the ASM Board of Directors.

### Reforms to governance

#### Harmonizing operating procedures with ASM bylaws

**Fig 1:**
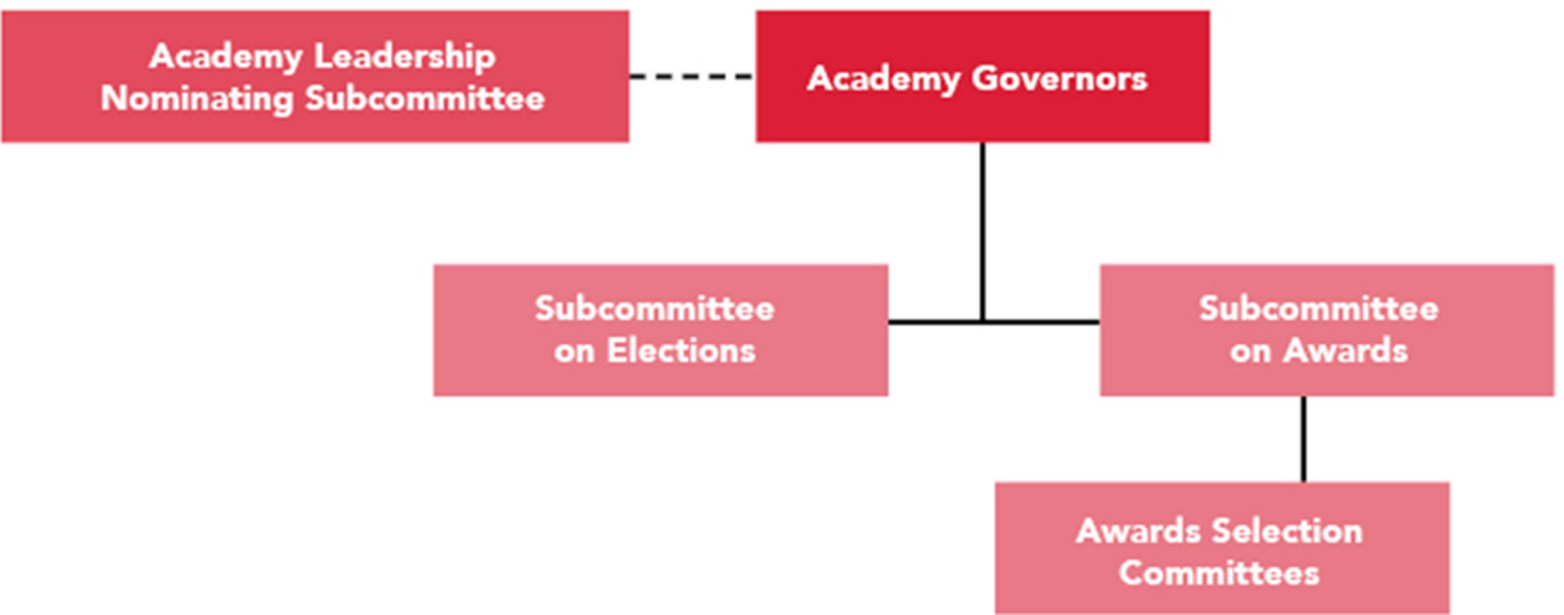
Academy governance structure. The Academy redefined its structure to align with the reform and published the information on the asm.org/academy website to inform the fellows about the roles and responsibilities of the Academy leadership in the spirit of transparency and accountability. Fellows in good standing are encouraged to put their names forward to be considered for Academy leadership.

No organization can have entities functioning with separate sets of bylaws. In 2018, Drs. Lynn Enquist and Timothy Donohue, an Academy Governor and ASM Board Member, respectively, undertook the task of transforming the AAM bylaws into a set of policies and procedures to reconcile them with the ASM bylaws. Figure 1 shows the Academy governance structure.

#### Renaming the Academy Board of Governors as the Committee of Academy Governors (or Academy Governors)

The reason for this change is that after the Academy was merged into ASM in 1969, there could only be one governing Board, which is currently known as the ASM Board of Directors. Other Academy committees then became subcommittees. Prior to the reforms, new Chairs and members of the Academy Governors were trained for their positions through an informal system whereby the departing chair/governor passed on instructions and suggestions. However, this system did not guarantee that the incoming Chairs or Academy Governors would have sufficient background information for a smooth transition. Hence, after the reform, a formal plan was put in place to provide the newly elected Academy leaders with an orientation to support them for the position. The Academy formalized the Chair-Elect position to be a full-year appointment prior to becoming the new Chair. Thus, after the election of 2022, Dr. Vanessa Sperandio joined the Governors as the Chair-Elect and had one full year to gain experience before assuming the leadership position in 2023.

#### Establishing triannual Academy Governor meetings

Prior to reforms, the Governors met only once per year, in a winter meeting where they approved the slate of newly elected fellows. However, a yearly meeting was not sufficient for the Governors to get to know one another, to work efficiently, or to address the new academy responsibilities (see below). Hence, two remote meetings, one in the spring and one in the fall, were added.

#### Establishing a mechanism for evaluating Academy Governors who are up for non-contested re-election prior to placement on the ballot

This mechanism was created for the unlikely situation that a Governor’s performance is deemed unsatisfactory. Under the prior system, the decision of individual Governors to serve a second term after a non-contested election was a decision that was left solely to the discretion of the individual Governor. Under the new mechanism, the unopposed election of Academy Governors running for a second term must be approved by the other Academy Governors who are not in that election cycle.

#### Developing a process to analyze and revoke, if needed, the Academy fellowship status of fellows with ethics violations

Cases involving individual fellows are referred to the ASM Ethics Committee following the ASM ethics review processes. Once the review is conducted, the ASM ethics committee makes a recommendation of whether the individual(s) meets the ASM ethics standard, and those recommendations are then reviewed and acted upon by the Academy Governors.

#### Embracing transparency in Academy leadership and information about fellows

Prior to the reforms, there was no information on the Academy Governors and Subcommittee members on the website. Today, the information is readily available on the Academy webpage, and one can easily search to find out whether an individual is a current Academy fellow in good standing.

#### Democratizing the Academy leadership election process

Since 2021, the Academy Governors charged the Academy Leadership Nominating Subcommittee to revise the Academy leadership election process. Today, any Academy fellow in good standing can put their name forward to be considered for an Academy leadership position.

### Reforms to election

The procedures and protocols for election to Academy fellowship were completely reformed, and these are described in reference 1. The most debated issue of the new protocols has been the limitation of each election class to 65 individuals. The rationale for capping each class at 65 can be found in reference 1. In 2023, the Academy Governors revisited the issue and considered the feedback from the fellows. After careful consideration, the Governors voted overwhelmingly to maintain the 65 limit for the next 3 years while continuing to collect data and input from the fellows for future review and decision.

### Reforms to communication

Prior to 2019, for a long period, many fellows did not receive information from the Academy and did not know how to get involved in the Academy after their election. The Academy recognized the need for more communication with its fellows, which includes more than 2,600 fellows who represent a significant proportion of the most accomplished scientists in microbiology and allied sciences. Hence, the Academy instituted a regular newsletter to inform fellows about new developments. The goal of the quarterly newsletter is to help bind fellows together through regular communications.

### Reforms to awards

With the responsibility of providing oversight of the ASM Awards and Prize Program, the Academy took steps to revise program web pages to ensure that information is well organized and clearly presented. The Academy also took steps to refine the nomination process to ensure that it is streamlined and less burdensome for the nominator. Finally, building on the effort of the Subcommittee on Awards in 2016 to restructure the ASM Awards, the Academy established a clear procedure to review and evaluate the ASM Awards on a regular basis to ensure the awards meet the needs of the diverse microbial science community and to help motivate the community to explore new scientific frontiers.

### Reforms to the mission and taking on climate change

**Fig 2:**
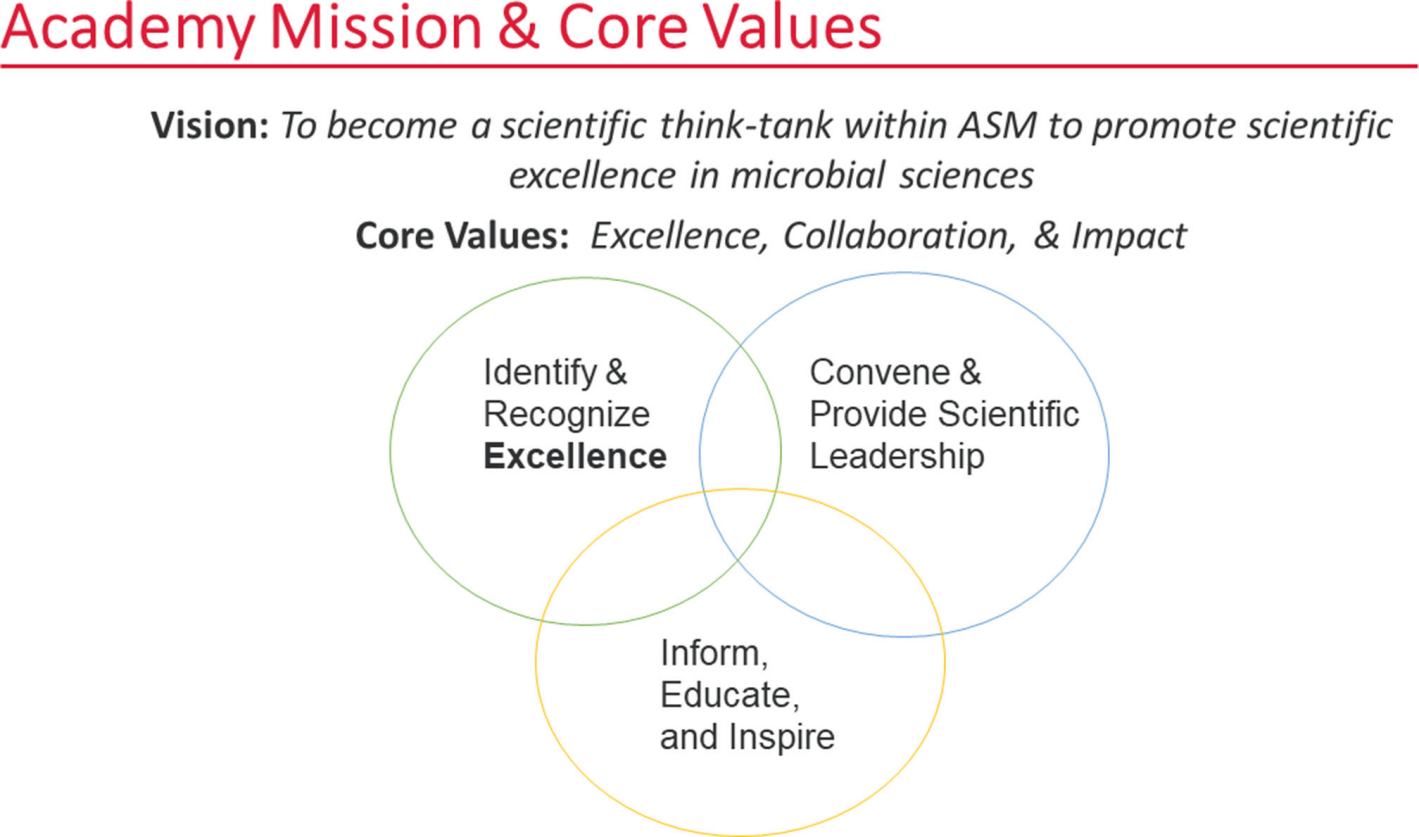
Academy’s renewed vision to become a scientific think-tank within ASM to better advance its mission to promote scientific excellence in microbial sciences. In 2021, the Academy Governors set the new vision and defined the core values to direct the Academy toward the path to deliver sustaining impacts to the field. The vision led to the establishment of the scientific portfolio, and climate change was selected to be the topic for the first portfolio.

Prior to 2019, the Academy was largely an honorific group that convened important colloquia on pressing topics, but did not focus on any specific field or issue. Since then it has evolved into a think tank for ASM (Fig. 2). In early 2021, the Academy Governors embraced a new strategic plan with a vision to become a scientific think tank within ASM (Fig. 2). With this vision, the Governors decided that the Academy should provide scientific leadership on pressing issues and polled its membership on topics to pursue. The aim was to choose one and then focus the resources of the Academy on a narrow set of questions and objectives where it could make a difference. In fact, the Academy, as a think-tank, can provide invaluable scientific input and analytical capabilities so that ASM programmatic and operational areas can collaboratively take on recommendations for action and implementation. The Academy fellows identified climate change, with its numerous connections to the microbial sciences, as a major concern. In 2021, the Academy established a 5-year scientific portfolio to “focus on promoting the understanding of the relationship between microbes and climate change and building a scientific framework to inform climate change policies and market innovations” (2). To launch the portfolio, the Academy invited Dr. James Tiedje to lead the inaugural colloquium on this topic on 5 November 2021 (3). Following this initial colloquium, the Academy established a task force composed of leading and diverse thinkers to further define specific areas where the portfolio could make a difference. Led by Dr. Jay Lennon, an Academy Governor, the task force identified three scientific focus areas: one health, biodiversity, and greenhouse gases. Furthermore, the colloquium rapidly identified a major concern in the fact that climate change models did not include microbial contributions, despite the fact that microbes are major producers and consumers of greenhouse gases and are responsible for a large proportion of carbon, oxygen, and nitrogen elemental flows in the biosphere (4). This revelation led to a follow-up virtual colloquium, held on 6 and 8 December 2022, that included both climatologists and microbiologists to identify which contributions from microbiology should be incorporated into climate modeling (5). The colloquium produced a list of 10 challenges that microbiologists and climate scientists must address as they collaborate. More importantly, the colloquium brought the two scientific communities closer, fostering dialogs and collaborations to fill scientific gaps. In addition, because of the Academy think-tank leadership, ASM hosted in 2023 a “guest track” at the annual ASM Microbe Meeting.

### Pandemic operations

The last in-person pre-COVID-19 pandemic meeting of the Academy Governors occurred in January 2020 at ASM Headquarters in Washington, DC. At the time, the SARS-CoV-2 outbreak was largely confined to China, but the virus began spreading worldwide. By March 2020, the virus was spreading rapidly in the USA, and the country went into shutdown of all non-essential operations. In close coordination with the ASM’s complex response to the pandemic, the Academy quickly undertook a key leadership role, establishing the COVID-19 Research Registry, a curated research collection of credible publications on this topic to help accelerate the scientific know-how at a moment of deep crisis. The effort was led by Dr. Lynn Enquist, an Academy Governor, along with Academy members Drs. Rozanne Sandri-Goldin and Vaughn Cooper, with advice from Dr. Harold Varmus. The Registry provided a tremendous resource for the scientific community during the pandemic to ensure that good science was promoted and brought forward. On 10 November 2020, the Academy, in partnership with the Council on Microbial Sciences (COMS), held a virtual symposium titled “Microbial Science Research in the Post-COVID Environment.” Although the pandemic was raging, and most academic activities had come to a standstill, there was a sense that science was changing rapidly and producing major results in record time. There was a need to begin to process the changes brought by the pandemic. The meeting suggested the need for a new value system that (i) emphasizes social impacts, (ii) rewards groups and not just individuals, and (iii) focuses on diversity, equity, and inclusion (6).

### Summary

The post-2019 reforms and pandemic experience have made the Academy stronger. The transformation of the Academy to become a scientific think tank with transparent and effective processes is highly critical for the future of the Academy. We believe that scientific institutions such as the Academy, with the capacity to lead efforts toward tackling major problems (such as the Academy’s focus in the climate change arena), are very important for the proper functioning of science and can significantly contribute to help strengthen society and evidence-based policies. This precedent of successful reforms establishes a foundation and a mechanism for further reforms as needed, possibly providing a path for future adaptations and harnessing and capitalizing on the inevitably new challenges that will present.
